# Mechanical stimuli such as shear stress and piezo1 stimulation generate red blood cell extracellular vesicles

**DOI:** 10.3389/fphys.2023.1246910

**Published:** 2023-08-30

**Authors:** Gurneet S. Sangha, Callie M. Weber, Ryan M. Sapp, Saini Setua, Kiruphagaran Thangaraju, Morgan Pettebone, Stephen C. Rogers, Allan Doctor, Paul W. Buehler, Alisa M. Clyne

**Affiliations:** ^1^ Fischell Department of Bioengineering, University of Maryland, College Park, MD, United States; ^2^ Department of Pediatrics, Center for Blood Oxygen Transport and Hemostasis, University of Maryland School of Medicine, Baltimore, MD, United States; ^3^ Department of Pathology, University of Maryland School of Medicine, Baltimore, MD, United States

**Keywords:** red blood cells, piezo1, shear stress, extracellular vesicles, exosomes

## Abstract

**Introduction:** Generating physiologically relevant red blood cell extracellular vesicles (RBC-EVs) for mechanistic studies is challenging. Herein, we investigated how to generate and isolate high concentrations of RBC-EVs *in vitro* via shear stress and mechanosensitive piezo1 ion channel stimulation.

**Methods:** RBC-EVs were generated by applying shear stress or the piezo1-agonist yoda1 to RBCs. We then investigated how piezo1 RBC-EV generation parameters (hematocrit, treatment time, treatment dose), isolation methods (membrane-based affinity, ultrafiltration, ultracentrifugation with and without size exclusion chromatography), and storage conditions impacted RBC-EV yield and purity. Lastly, we used pressure myography to determine how RBC-EVs isolated using different methods affected mouse carotid artery vasodilation.

**Results:** Our results showed that treating RBCs at 6% hematocrit with 10 µM yoda1 for 30 min and isolating RBC-EVs via ultracentrifugation minimized hemolysis, maximized yield and purity, and produced the most consistent RBC-EV preparations. Co-isolated contaminants in impure samples, but not piezo1 RBC-EVs, induced mouse carotid artery vasodilation.

**Conclusion:** This work shows that RBC-EVs can be generated through piezo1 stimulation and may be generated *in vivo* under physiologic flow conditions. Our studies further emphasize the importance of characterizing EV generation and isolation parameters before using EVs for mechanistic analysis since RBC-EV purity can impact functional outcomes.

## 1 Introduction

Red blood cells (RBCs) are exposed to both chemical and physical stresses as they circulate throughout the body. Perhaps in response to these stresses, RBCs lose 20% of their volume during their 120-day lifecycle (Werre et al., 2004; Bosman, 2013). Some RBC volume loss comes in the form of extracellular vesicles (RBC-EVs), defined as lipid-bound particles 40–1,000 nm in diameter that transport signaling molecules (e.g., proteins, metabolites, lipid molecules) to other cells (Van Niel et al., 2018; Thangaraju et al., 2020). RBC-EVs affect vascular health and disease. RBC-EVs generated in pathological conditions such as obstructive sleep apnea (Peng et al., 2020), sickle cell disease (Jana et al., 2018; Gemel et al., 2020; Lapping-Carr et al., 2020), or blood storage (Straat et al., 2016; Almizraq et al., 2018) exacerbate endothelial dysfunction, the initial hallmark of vascular disease. The physiological function of RBC-EVs is unclear, as most published studies focus on RBC-EVs generated in pathological conditions.

RBC-EVs may be generated during physiological shear stress as RBCs (8–10 µm diameter) squeeze through capillaries (2–4 µm diameter). Shear stress could trigger RBC-EV production by stimulating piezo1, a mechanosensitive Ca^2+^ ion channel (Cahalan et al., 2015; Gottlieb, 2017; Parpaite and Coste, 2017). A seminal study by Larsen et al. (1981) showed that shear stress enhances RBC Ca^2+^ permeability. Danielczok et al. (2017) later confirmed that RBCs squeezing through capillaries transiently increase intracellular Ca^2+^ through piezo1 activation. RBC Ca^2+^ uptake activates scramblase, a transmembrane protein that translocates RBC phosphatidylserine from the inner to outer plasma membrane leaflet to trigger RBC-EV release (Gonzalez et al., 2009; Nguyen et al., 2016; Wesseling et al., 2016). Piezo1 stimulation may also generate RBC-EVs through similar Ca^2+^-dependent mechanisms. However, it remains unclear how RBC-EVs generated by physiological shear stress or piezo1 stimulation affect endothelial function and subsequent vascular health.

RBC-EVs are also a promising drug delivery vehicle for pharmaceutical, small molecule, and gene editing tools (Usman et al., 2018; Chiangjong et al., 2021). Emerging drug delivery methods rely on immunogenic or cytotoxic viruses, lipid transfection reagents, and lipid nanoparticles (Usman et al., 2018). RBC-EVs are a promising choice to transport therapeutic molecules because they lack nuclear and mitochondrial DNA, can be readily generated from the patient’s RBCs, and are easily generated in large quantities. However, RBC-EVs are also bioactive, containing cytosolic and membrane-bound signaling molecules that can affect physiological function (Chiangjong et al., 2021). Therefore, it is important to understand how RBC-EV contents affect health and disease prior to developing RBC-EV-based drug delivery therapies. We identify vesicles as EVs throughout the paper to avoid misidentification of the types of vesicles (e.g., exosomes, ectosomes, microvesicles), following the latest MISEV’s recommendations (Théry et al., 2018).

Isolating pure RBC-EVs is challenging. RBC-EVs can be isolated from human blood; however, these samples are difficult to purify since they contain EVs from multiple cell types, as well as non-EV contaminants such as large protein aggregates and lipoproteins (Webber and Clayton, 2013; Johnsen et al., 2019). Purer RBC-EVs can be produced *in vitro* by treating RBCs with ionophore to stimulate vesiculation via Ca^2+^-dependent mechanisms (Allan et al., 1980; Salzer et al., 2002; Nguyen et al., 2016; Usman et al., 2018; Bigdelou and Farnoud, 2020), *tert*-butyl hydroperoxide to induce oxidative stress (Sudnitsyna et al., 2020), oxygen cycling to mimic RBC hypoxia (Peng et al., 2020), or long-term storage to produce RBC-EVs relevant in blood transfusions (Straat et al., 2016; Almizraq et al., 2018). In a few studies, RBC-EVs were generated via shear stress from needle or high-pressure extrusion (Jy et al., 2013; Jana et al., 2018). Just as there are many methods to generate RBC-EVs, there are also many methods to isolate RBC-EVs, including membrane-based affinity, ultrafiltration, and ultracentrifugation. The techniques used to produce and isolate EVs can impact RBC-EV concentration, contents, and purity, and therefore the biological function of these RBC-EVs.

RBC-EVs generated by shear stress are of particular interest because they may serve as conduits for intercellular signaling throughout the body; however, the lack of standard methods for generating, isolating, and storing mechanically-stimulated RBC-EVs hinders biological studies. Therefore, we tested how RBC mechanical stimulation (shear stress, piezo1), EV generation parameters (hematocrit, treatment time, treatment concentration), EV isolation technique (membrane-based affinity, ultrafiltration, ultracentrifugation), and storage (temperature, freeze-thaw, storage time) impacted RBC-EV concentration, size, and purity. Our methods will guide investigators in generating, isolating, and storing RBC-EVs and promote the thorough characterization of EVs from varied cell types.

## 2 Materials and methods

### 2.1 Human participants

All procedures and documents were approved by the University of Maryland Institutional Review Board (IRB Net ID: 1385293-7) and conformed to the Declaration of Helsinki. Twelve healthy subjects (*n* = 6 men, *n* = 6 women) between the ages of 20 and 31 years old were recruited for up to eight blood draws. All participants provided written informed consent and completed a health history questionnaire. Exclusion criteria included the following: current or prior metabolic or cardiovascular disease, cancer, current or previous smoker, use of potential study-confounding medications, anemia, or body weight <110 pounds.

### 2.2 Blood collection

Participants reported to the laboratory in the morning following an overnight fast of ≥10 h. Prior to each blood draw, participants also refrained from using alcohol, non-steroidal anti-inflammatory drugs (NSAIDS), and medications for 24 h, as well as participating in physical exercise for at least 16 h. Participants were allowed to continue taking regular oral contraceptives. Additionally, all participants reported not being sick for at least 1 week prior to blood draws. After a seated rest of ∼5 min, 20–30 mL of blood was drawn from an antecubital vein into tubes (BD) containing acid citrate dextrose (ACD) as an anticoagulant. Blood samples were immediately placed on ice until RBC isolation (usually <1 h).

### 2.3 RBC isolation

Blood was pooled from two male and two female subjects and centrifuged at ×1,500 g for 5 min. Plasma was then removed, and RBCs were washed four times with phosphate buffered saline (PBS; Thermo Fisher Scientific) to remove the buffy coat (white blood cells and platelets; Supplementary Figure S1). For all experiments, RBCs were resuspended in PBS supplemented with 99 mg/dL glucose and 1 mM calcium chloride (CaCl_2_) at pH 7.4. All experiments were performed at 21% oxygen (room air).

### 2.4 RBC viability assay

To ensure that RBC were viable before and after EV generation, RBC (5 million cells/mL) were incubated with 2 μg/mL calcein AM (Thermo Fisher Scientific) for 15 min at 37°C. RBC were then streaked onto a glass slide, sandwiched with a glass coverslip, and imaged at ×20 on a Revolve fluorescent microscope (Echo). RBC viability was determined based on co-localization of calcein AM with cells (by brightfield microscopy) in ImageJ (NIH). Percent viability was calculated as viable RBCs divided by the number of particles.

### 2.5 Hemolysis measurement

Hemolysis was calculated from the hemoglobin absorbance of 200 µL RBC supernatant at 541 nm using a Spark multimode microplate reader (Tecan) (Zhang et al., 2016; Nazemidashtarjandi and Farnoud, 2019; Bigdelou and Farnoud, 2020). Absorbance was used to calculate hemolysis as: (Nazemidashtarjandi and Farnoud, 2019; Bigdelou and Farnoud, 2020):

$$\%Hemolysis=\left(\frac{A_{T}-A_{o}}{A_{100}-A_{o}}\right)x\;100$$

Where A_0_ is the supernatant absorbance from RBCs in PBS (0% cell lysis), A_100_ is the supernatant absorbance from RBCs in deionized water (100% cell lysis), and A_T_ is the supernatant absorbance from experimentally treated RBCs (e.g., DMSO, yoda1, Ca^2+^ ionophore).

### 2.6 RBC-EV production

RBC-EVs were mechanically stimulated using a rheometer equipped with a double gap concentric cylinder (MCR 320, Anton Paar) that enabled precise shear stress exposure. We exposed 50 mL of 6% hematocrit to shear stress (250 dynes/cm^2^) for 10 min to stimulate RBC-EV release. RBCs in static conditions served as controls. Shear stress-induced RBC-EVs were then concentrated from 50 mL to 400 µL using Amicon Ultra-15 centrifugal filter units (UFC910024, Millipore Sigma; 100 kDa molecular weight cut off).

RBC-EVs were also generated by treating RBCs with piezo1-agonist yoda1, Ca^2+^ ionophore, or 0.28% dimethyl sulfoxide (DMSO; vehicle control) in a 5% CO_2_, 37°C incubator. Yoda1, Ca^2+^ ionophore, and DMSO were purchased from Sigma-Aldrich. The RBC treatment protocol was tuned to improve piezo1 RBC-EV yield and purity using 1, 3, 6, 18% hematocrit; 0.5, 1, 2, and 24 h yoda1 treatment times; and 1, 10, 100 µM yoda1 concentration. Ca^2+^ ionophore RBC-EVs, used as a gold-standard comparison, were produced with 6% hematocrit, 1 or 24 h treatment times, and 10 or 100 µM Ca^2+^ ionophore concentration.

### 2.7 RBC-EV isolation

Plasma EVs were isolated from healthy donors using ultracentrifugation method. Cell debris and apoptotic bodies were removed by differential centrifugations (×1,500 g for 15 min, ×3,200 g for 15 min and followed by ×10,000 g for 30 min at 4°C). The plasma supernatants were diluted with PBS and passed through a 0.22 µM polyethersulfone syringe filter. The filtrates were centrifuged again at ×100,000 g for 70 min and the final plasma EV pellet were resuspended in 500 µL PBS.

RBC-EVs generated *in vitro* were isolated using membrane-based affinity binding, ultrafiltration, or ultracentrifugation (JA-30.50 Ti; Beckman Coulter; Table 1). We used the exoEasy Maxi Kit (Qiagen) as our representative membrane-based affinity binding method and Amicon Ultra-15 centrifugal units (Sigma-Aldrich) as our representative ultrafiltration method. For all RBC-EV isolation methods, RBC and cell debris were first removed by differential centrifugation at 600 *g* for 20 min, ×1,600 g for 15 min, and ×3,250 g for 15 min at room temperature, followed by ×10,000 g for 30 min at 4°C. The RBC supernatant was then passed through a 0.22 µM polyethersulfone syringe filter (371-2115-OEM; Foxx Life Sciences) for ultracentrifugation and Amicon Ultra-15 isolations or a 0.8 µM surfactant-free cellulose acetate syringe filter (16,592, Sartorius) for exoEasy isolations, as per manufacturer recommendation. For membrane-based affinity binding, 8 mL RBC supernatant and 8 mL XBP buffer were mixed and passed through the exoEasy affinity spin column to bind RBC-EVs to the membrane. RBC-EVs were then eluted using 500 µL XE buffer per manufacturer instructions. For ultrafiltration, 12 mL RBC supernatant was passed through an Amicon Ultra-15 centrifugal filter at ×3,260 g for 15 min. A second Amicon Ultra-15 centrifuge spin was used to wash and concentrate RBC-EVs in 2X PBS. For ultracentrifugation, 35 mL RBC supernatant was centrifuged at ×100,000 g for 70 min at 4°C. The RBC-EV pellet was washed in PBS, centrifuged again at ×100,000 g for 70 min, and finally resuspended in 400 µL 2X PBS. RBC-EVs were stored at −80°C until use; therefore, all RBC-EVs in this study experienced one freeze-thaw cycle.

**TABLE 1:** RBC-EV isolation protocol summary.

	Ultracentrifugation		Ultrafiltration (Amicon Ultra-15)		Membrane-based affinity (ExoEasy Maxi kit)	
Starting volume	35 mL		12 mL		8 mL	
Cell debris removal	×600 g for 20 min					
	×1,600 g for 15 min					
	×3,260 g for 15 min					
	×10,000 g for 30 min					
	Pass supernatant through 0.22 µm filter		Pass supernatant through 0.22 µm filter		Pass supernatant through 0.8 µm filter	
EV Isolation	×100,000 g for 70 min at 4°C		×3,820 g for 15 min at 25°C		RBC-EV Sample + Buffer XBP ×500 g for 1 min	
	×100,000 g for 70 min at 4°C		×3,820 g for 15 min at 25°C		Buffer XWP wash ×5,000 g for 5 min	
					Buffer XE elution ×500 g for 5 min	
Purification	− SEC	+ SEC	− SEC	+ SEC	− SEC	+ SEC

### 2.8 Size exclusion chromatography (SEC)

A subset of RBC-EVs isolated using membrane-based affinity binding, ultrafiltration, or ultracentrifugation were further purified using SEC. 500 µL RBC-EV sample was added to SEC columns (qEV original, IZON) and eluted with 2X PBS. Six 500 µL eluate fractions were collected. Protein concentration was measured to identify fractions containing RBC-EVs by spectrophotometrically reading absorbance at 280 nm (NanoDrop 2000c; Thermo Fisher Scientific).

### 2.9 EV size and concentration

Tunable resistive pulse sensing (TRPS) was used to quantify RBC-EV concentration and size (50–330 nm) using a qNano Gold (IZON Science) with NP100 nanopore membranes. RBC-EV samples were first filtered through 0.22 µm ultrafree-MC tubes (UFC30GV, Millipore Sigma) to remove large particles that could clog the nanopore. TRPS voltage was set between 0.5–0.7 V to achieve a stable 130 nA current. Particles were counted when root mean square (RMS) noise was below 10 pA, and the particle rate was linear. At least 500 particles were counted at particle rates between 200-1,500 particles/minute and 5 or 10 mBar pressure. TRPS was calibrated using CPC100 beads (mean diameter: 100 nm).

### 2.10 Transmission electron microscopy (TEM)

TEM was used to confirm RBC-EV size and visualize RBC-EV shape. We applied 5 µL RBC-EV sample to a 200 mesh copper grid coated with formvar carbon film (FCF200-Cu, Electron Microscopy Sciences) for 30 s. The copper grid was washed with PBS and stained using 5 µL 2% uranyl acetate for 30 s. TEM images were acquired using a Hitachi HT7700 Transmission Electron Microscope.

### 2.11 Western blot

Western blot was used to detect RBC and RBC-EV proteins. RBCs were lysed with RIPA buffer (Thermo Fisher Scientific) containing Halt Protease Inhibitor Cocktail (Thermo Fisher Scientific) and EDTA (Thermo Fisher Scientific). Lysates were centrifuged for 15 min at 4°C to remove cellular debris. Lysate protein was quantified using a BCA assay (Thermo Fisher Scientific). We loaded 200 µg RBC protein lysate into each well of a NuPAGE 4%–12% Bis-Tris gel (Thermo Fisher Scientific) for protein separation by SDS-PAGE. RBC-EVs were lysed by sonicating 3 times for 15 s each. We then loaded 29 µL of lysed RBC-EVs in NuPAGE 4%–12% Bis-Tris protein gels. Separated proteins were transferred onto polyvinylidene difluoride (PVDF) membranes using an iBlot 2 (Thermo Fisher Scientific) semi-dry transfer system. PVDF membranes were blocked in 5% bovine serum albumin (BSA) in TBS-Tween (TBS-T) for 1 h at room temperature. Blots were then incubated with primary antibodies in 1% BSA in TBS-T overnight at 4°C. Primary antibodies included ALIX (1:500; sc-53538), TSG101 (1:500; sc-7964), CD9 (1:500; sc-13118), CD63 (1:500; sc-5275), calnexin (1:500; sc-23954), stomatin (1:500; sc-376869), CD52 (1:500; sc-51560), and GAPDH (1:1,000; 2118S; all from Cell Signaling Technology). Blots were then incubated with the appropriate mouse or rabbit horseradish peroxidase conjugated secondary antibody (Thermo Fisher Scientific) for 2 h at room temperature. Protein bands were detected using a chemiluminescence kit (SuperSignal West Pico PLUS, Thermo Fisher Scientific) and imaged immediately using an Alpha Innotech Fluorchem Imager. ImageJ was used to quantify band intensity, normalized to GAPDH.

### 2.12 Flow cytometry

Flow cytometry was used to classify the cellular origin of isolated EVs based on surface markers (Setua et al., 2022). EVs were labeled for 45 min at room temperature in the dark with Alexa Fluor 647 conjugated anti-CD63 (Clone: H5C6; 353,015), BV421 conjugated anti-CD41 (Clone: HIP8; 303,729), PE dazzle 594 conjugated anti-CD235a (Clone: HI264; 349,119) and BV605 conjugated anti-CD31 (Clone: WM59; 303,121; all from Biolegend). EVs were then resuspended in 500 μL particle free PBS and analyzed using a BD FACSAria Flow cytometer (Becton Dickenson), which had been previously calibrated using a flow cytometry sub-micron particle size reference kit (F13839; ThermoFisher). Data were analyzed using FlowJo 10.8.0 software (Becton Dickenson). Buffer and unlabeled EVs were the negative controls. Forward scatter (FSC) and side scatter (SSC) were set to gate the EV population. Each marker expression was represented by histogram and normalized to mode. 200 nm calibration beads were used for FSC and SSC parameters.

### 2.13 Protein contamination

RBC-EV protein contamination was measured using the particle/total protein ratio (particles/µg protein). (Webber and Clayton, 2013). RBC-EV concentration was calculated using TRPS. Sample protein concentration was quantified using a Bicinchoninic acid (BCA) protein assay kit (Thermo Fisher Scientific), a colorimetric method to quantify protein, using BSA standards ranging from 20-2000 μg/mL.

### 2.14 Protein aggregates

Protein aggregates (non-EV contaminants) were quantified using the EV/Non-EV particle rate ratio. Non-EV samples were created by vortexing samples in 1% Triton-X 100 surfactant every 15 min for 1 h to lyse EVs (Tian et al., 2020). Particle rate was then measured in lysed and intact RBC-EV samples by counting particles via TRPS over 1 min. Particle rate was used as a surrogate for concentration because a particle rate of 100 particles/min is needed to accurately quantify size and concentration, and lysed samples never reached this particle rate.

### 2.15 Animals

The animal protocol was approved by the University of Maryland Institutional Animal Care and Use Committee (IRBNet ID: 1664120-1). 12-week-old male C57Bl/6 mice were acquired from Jackson Laboratory (Bar Harbor, ME) and maintained on a normal chow diet *ad libitum*. Animals were acclimated for at least 3 days in the University of Maryland vivarium prior to euthanasia by exsanguination. The carotid arteries were quickly excised and placed in cold HEPES physiological saline solution (HEPES-PSS) and used within 24 h. HEPES-PSS (1 L DI water, 6.96 g NaCl, 0.35g KCl, 0.29 g MgSO_4_*7H_2_O, 0.16 g KH_2_PO_4_, 0.31 g CaCl_2_*2H_2_O, 1.25 g NaHCO_3_, 2.38 g HEPES, 0.99 g glucose, 2 mL 500x EDTA and pH 7.4) was made fresh on the experiment day.

### 2.16 Pressure myography

Carotid arteries were cannulated with stainless steel micropipettes in a pressure myograph chamber (114P; DMT) containing cold HEPES-PSS. The myograph chamber was then slowly warmed to 37°C and aerated with carbogen (5% CO_2_, 95% O_2_). Arterial pressure was increased in 10 mmHg increments, every 5 min, up to 40 mmHg. Carotid arteries were then equilibrated at 50 mmHg for an additional 15 min. Vessel viability was confirmed by superfusing arteries with high potassium PSS (KPSS; 250 mL DI water, 3.69 g NaCl, 18.6 g KCl, 0.36 g MgSO_4_*7H_2_O, 0.41 g KH_2_PO_4_, 0.46 g CaCl_2_*2H_2_O, 0.21 g NaHCO_3_, 0.18 g glucose, 95 µL 500x EDTA) for 5 min. Carotid arteries were considered viable if the vessel constricted at least 10%. KPSS tests were repeated two times. The carotid arteries were then washed with HEPES-PSS and submaximally constricted 10%–15% using 10^−6^ M phenylephrine (207240100; Acros Organics). Vasodilation was measured by treating carotid arteries for 5 min with RBC-EVs isolated using membrane-based affinity, ultrafiltration, and ultracentrifugation. Finally, endothelial-dependent vasodilation was measured in the same carotid arteries using an acetylcholine dose-response ranging from 10^−9^ to 10^−5^ M acetylcholine. Vasodilation was measured using the maximum steady-state diameter (D_a_), steady-state diameter after phenylephrine preconstruction (D_p_), and maximal steady-state baseline vessel diameter (D_m_).

$$Vasodilation\;\left(\%\right)=\left[\frac{D_{a}-D_{p}}{D_{m}-D_{p}}\right]x\;100$$

### 2.17 Hemoglobin ELISA

RBC-EV hemoglobin concentration was measured using a hemoglobin ELISA (Thermo Fisher Scientific) as per manufacturer protocol. RBC-EVs were diluted ×250,000 with the appropriate assay diluents. Samples were assayed in duplicate. Immediately after the addition of stop solution, absorbance was measured at 450 nm in a Tekan Spark Multimode Microplate Reader.

## 3 Results

### 3.1 Healthy humans have circulating RBC-EVs

**FIGURE 1:**
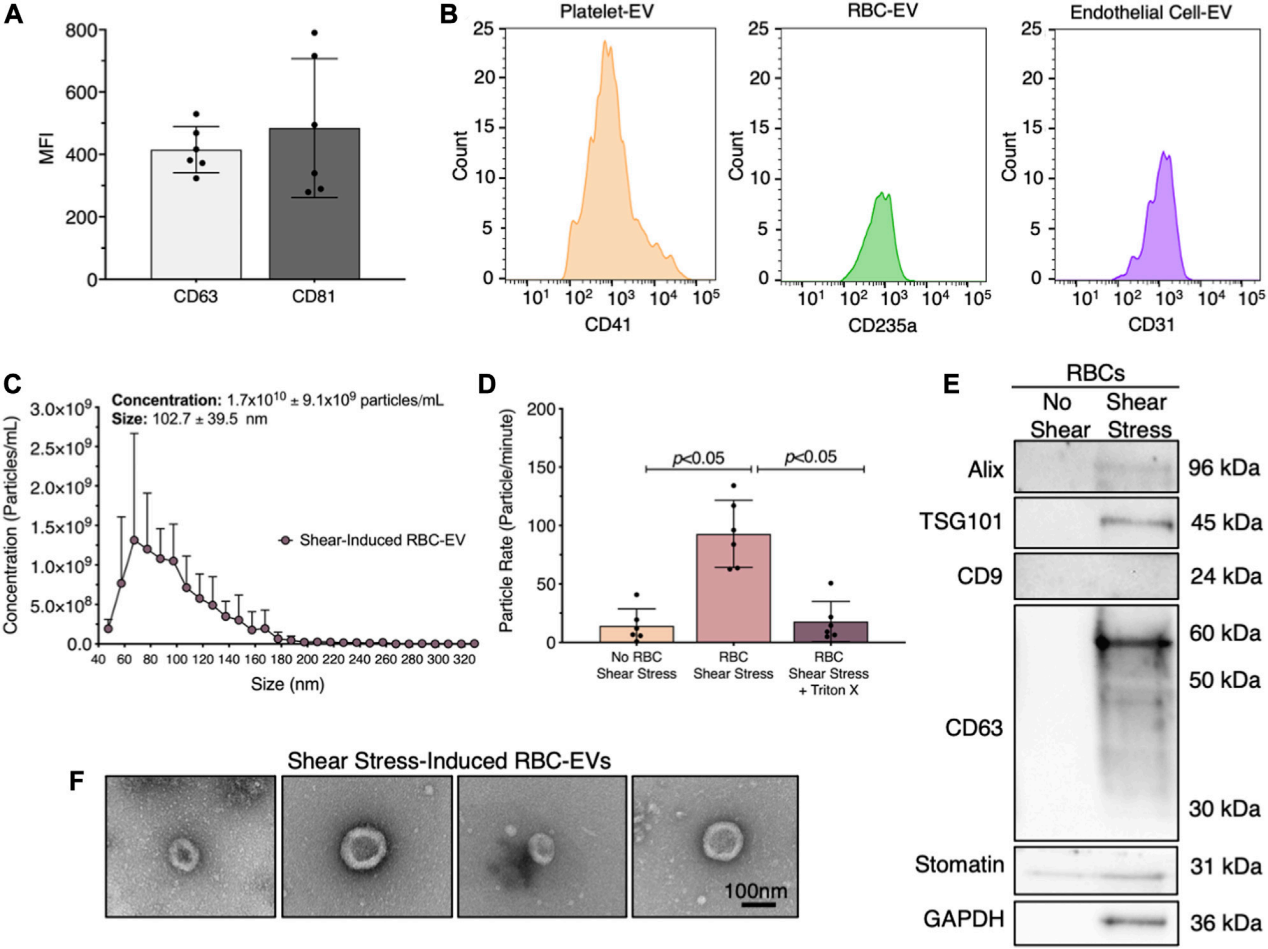
RBC-EVs circulate in healthy humans, and particles released by RBCs exposed to shear stress contain EVs. **(A)** Flow cytometry mean fluorescence intensity (MFI) of EV markers (CD63^+^ and CD81^+^) on particles isolated from plasma by ultracentrifugation. **(B)** Flow cytometry histogram of plasma EVs for platelets (CD41^+^), RBCs (CD235a^+^), and endothelial cells (CD31^+^). **(C)** Size histogram of particles isolated from sheared RBCs using ultrafiltration. **(D)** Particle rate of samples isolated from RBCs in static conditions, RBC exposed to shear stress, and RBC exposed to shear stress with isolated particles treated with 1% Triton X-100. Particle rate was quantified by measuring the number of particles that passed through the TRPS nanopore over 1 min. **(E)** Representative Western blot of particles isolated from RBC exposed to shear stress for cytoplasmic EV markers ALIX and TSG101, surface EV markers CD9 and CD63, RBC-specific marker stomatin, and housekeeper GAPDH. **(F)** Representative TEM images of particles generated by exposing RBC to shear stress. For flow cytometry, each data point represents plasma from one person, with the experiment repeated six times (*n* = 6). For all other studies, each data point represents a sample in which RBCs were pooled from four people. Shear stress studies were performed in singlicate and repeated six times (*n* = 6). Western blots were run in triplicate and repeated two times (*n* = 6 per condition). TEM images were acquired from two samples (*n* = 2 per condition). Data are represented as mean ± standard deviation. Statistical significance for panel d was determined using Kruskal–Wallis with Dunn’s multiple comparison test.

We first demonstrated that healthy human plasma contains RBC-EVs. EVs were isolated from human plasma using ultracentrifugation and flow cytometry was used to confirm that the isolated particles contain EV markers CD63 and CD81 (Figure 1A). We then labeled the EVs for their cellular source, including platelet (CD41^+^), RBCs (CD235a+), and endothelial cell (CD31^+^) (Figure 1B). While platelet EVs composed the highest percentage of plasma EVs, endothelial and RBC-EVs also greatly contributed to the plasma EV pool.

### 3.2 Shear stress triggers RBC-EV release

Next, we studied whether RBCs exposed to shear stress produce EVs. We sheared RBCs at 250 dynes/cm^2^ using a rheometer equipped with a double gap concentric cylinder, concentrated shear stress-induced particles by ultrafiltration (Amicon Ultra-15), and quantified particle concentration and size using TRPS. Sheared RBCs generated 1.7 × 10^10^ ± 9.1 × 10^9^ particles/mL, with particle sizes ranging from 63 to 143 nm (Figure 1C). The particle rate from sheared RBCs was 6.6-fold higher than the particle rate from RBCs in static conditions (*p* < 0.05; Figure 1D). To demonstrate that these particles were lipid-bound EVs, we treated the samples with 1% Triton X-100, which lyses EVs, and counted the remaining particles. Particle rate significantly decreased when sheared RBC samples were treated with Triton X-100 (Figure 1D). We further confirmed by Western blot that the shear stress-induced RBC particles contained cytoplasmic EV markers ALIX and TSG101 and surface EV marker CD63, as well as RBC-specific marker stomatin (Figure 1E). Particles isolated from RBCs that did not undergo shear stress did not contain any measurable EV markers. Finally, we used TEM to demonstrate that the shear stress-generated RBC particles resembled EVs and were ∼100 nm in diameter (Figure 1F). We were unable to find any particles that resembled EVs in samples from RBC in static conditions. We therefore concluded that shear stress generated RBC-EVs.

### 3.3 Piezo1 stimulation produced RBC-EVs with minimal loss of RBC viability and low hemolysis

**FIGURE 2:**
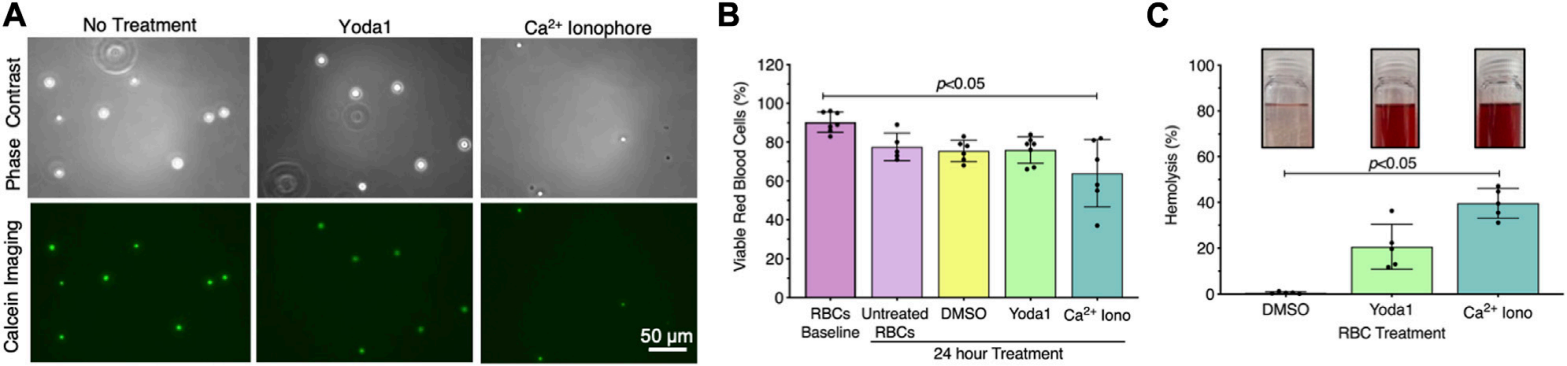
RBCs treated with piezo1-agonist yoda1 were viable with minimal hemolysis. **(A)** Representative calcein-labeled (green) RBC fluorescent microscopy images, with **(B)** quantification to assess RBC viability. **(C)** RBC hemolysis, as measured by hemoglobin absorbance at 541 nm in the RBC supernatant. Insets show representative images of RBC supernatant after treatment. RBCs were treated with 0.28% DMSO (vehicle control), 100 µM yoda1, or 100 µM Ca^2+^ ionophore (iono) for 24 h. Each data point represents a sample in which RBCs were pooled from four people. Cell viability studies were performed in singlicate and repeated five to seven times (*n* = 5–7 per condition). Hemolysis studies were performed in singlicate and repeated five times (*n* = 5 per condition). Data are represented as mean ± standard deviation. Statistical significance for panels b and c was determined using Kruskal–Wallis with Dunn’s multiple comparison test.

Exposing RBCs to shear stress is labor intensive, time-consuming, and requires specialized equipment that is not accessible to all investigators. Therefore, we investigated whether we could generate RBC-EVs by chemically stimulating the mechanosensitive ion channel piezo1 via the agonist yoda1 in the presence of Ca^2+^. Yoda1 treatment was compared to Ca^2+^ ionophore treatment, as Ca^2+^ ionophore is the standard published method for RBC-EV generation (Allan et al., 1980; Salzer et al., 2002; Nguyen et al., 2016; Usman et al., 2018; Bigdelou and Farnoud, 2020). We first confirmed that yoda1 treatment did not impact RBC viability. Untreated RBCs were ∼90% viable after 24 h incubation at 37°C, as determined by calcein labeling (Figure 2A). Approximately 80% of RBCs were viable after 24 h of treatment with 100 µm yoda1, similar to 0.28% DMSO (vehicle control). RBC viability significantly decreased to 64% ± 17% with 100 µm Ca^2+^ ionophore treatment (*p* < 0.05 compared to freshly isolated RBCs; Figure 2B). Ca^2+^ ionophore-treated RBCs also experienced greater hemolysis (Figure 2C) and produced more cell debris (Supplementary Figure S2) than yoda1-treated RBCs. We did not find statistical significance when comparing viability (Figure 2B; *p* > 0.99) and hemolysis (Figure 2C; *p* = 0.41) between yoda1-and Ca^2+^ ionophore-treated RBCs.

**FIGURE 3:**
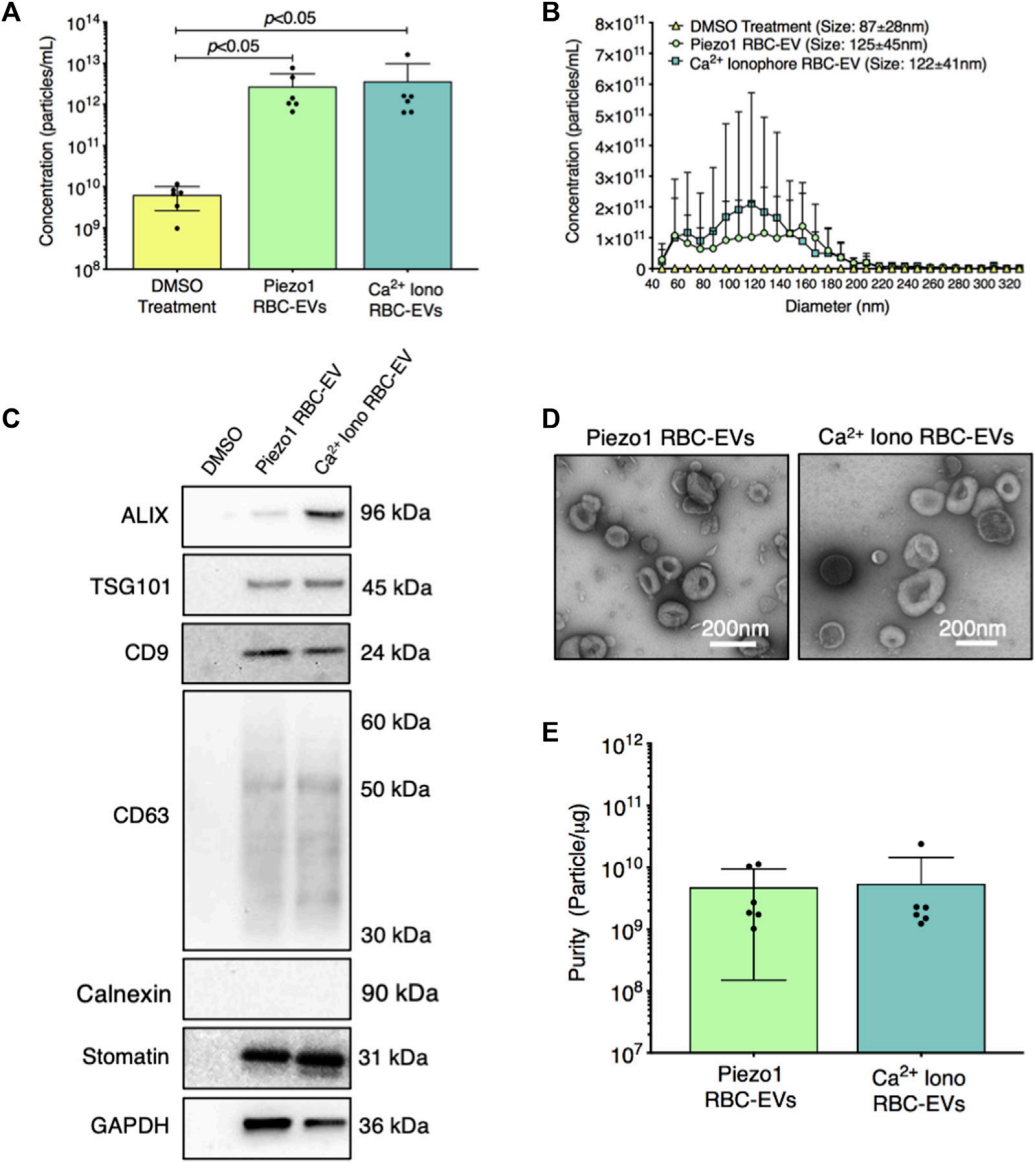
RBC particles generated through piezo1 stimulation were confirmed as RBC-EVs by size, protein markers, and morphology. **(A)** Particle concentration in RBC supernatant after 0.28% DMSO (vehicle control), 100 µM yoda1, and 100 µM Ca^2+^ ionophore (iono) treatment for 24 h, as measured by TRPS **(B)** Size histogram of particles isolated from RBC supernatant after DMSO, yoda1, and Ca^2+^ ionophore treatment, as measured by TRPS **(C)** Representative Western blots of cytoplasmic EV markers ALIX and TSG101, surface EV markers CD9 and CD63, apoptotic body marker calnexin, RBC-specific marker stomatin, and housekeeper GAPDH. **(D)** Representative TEM images of piezo1 and Ca^2+^ ionophore RBC-EVs. **(E)** Piezo1 and Ca^2+^ ionophore RBC-EV purity, as measured by particles/µg total protein. Each data point represents a sample in which RBCs were pooled from four people. TRPS and purity studies were performed in singlicate and repeated six times (*n* = 6 per condition). Western blots were performed in triplicate and repeated two times (*n* = 6 per condition). TEM images were acquired from two samples (*n* = 2 per condition). Data are represented as mean ± standard deviation. Statistical significance for panel a was determined using Kruskal–Wallis with Dunn’s multiple comparison test.

We then established if yoda1 treatment generated RBC-EVs. When RBCs were treated with 100 µM yoda1 for 24 h, the supernatant contained 2.7 × 10^12^ ± 2.8 × 10^12^ particles/mL with a diameter of 125 ± 45 nm (Figures 3A, B). Ca^2+^ ionophore treatment (also 100 µM for 24 h) produced 3.6 × 10^12^ ± 6.2 × 10^12^ particles/mL with a similar size (122 ± 41 nm diameter; Figures 3A, B). DMSO vehicle control produced ×1,000 fewer particles (6.3 × 10^9^ ± 3.7 × 10^9^ particle/mL) with a smaller size (87 ± 28 nm diameter; Figures 3A, B). Both piezo1-and Ca^2+^ ionophore-generated RBC particles contained cytoplasmic EV markers ALIX and TSG101 and surface EV markers CD9 and CD63, along with RBC-specific marker stomatin (Figure 3C). ALIX quantity was consistently greater in Ca^2+^ ionophore as compared to piezo1 RBC particles (*p* > 0.05; Supplementary Figure S3). We did not detect apoptotic body marker Calnexin in piezo1 or Ca^2+^ ionophore-generated RBC particles (Figure 3C). TEM confirmed that piezo1 and Ca^2+^ ionophore produced RBC particles that resembled EVs and were similar in size and morphology (Figure 3D). Finally, piezo1 and Ca^2+^ ionophore RBC-EVs had similar purity, as determined by the particle/total protein ratio (Figure 3E). These data confirmed that RBC treatment with yoda1 generated RBC-EVs.

### 3.4 RBC-EV concentration and purity were maximized by treating 6% hematocrit with 10 µM yoda1 for 30 min

**FIGURE 4:**
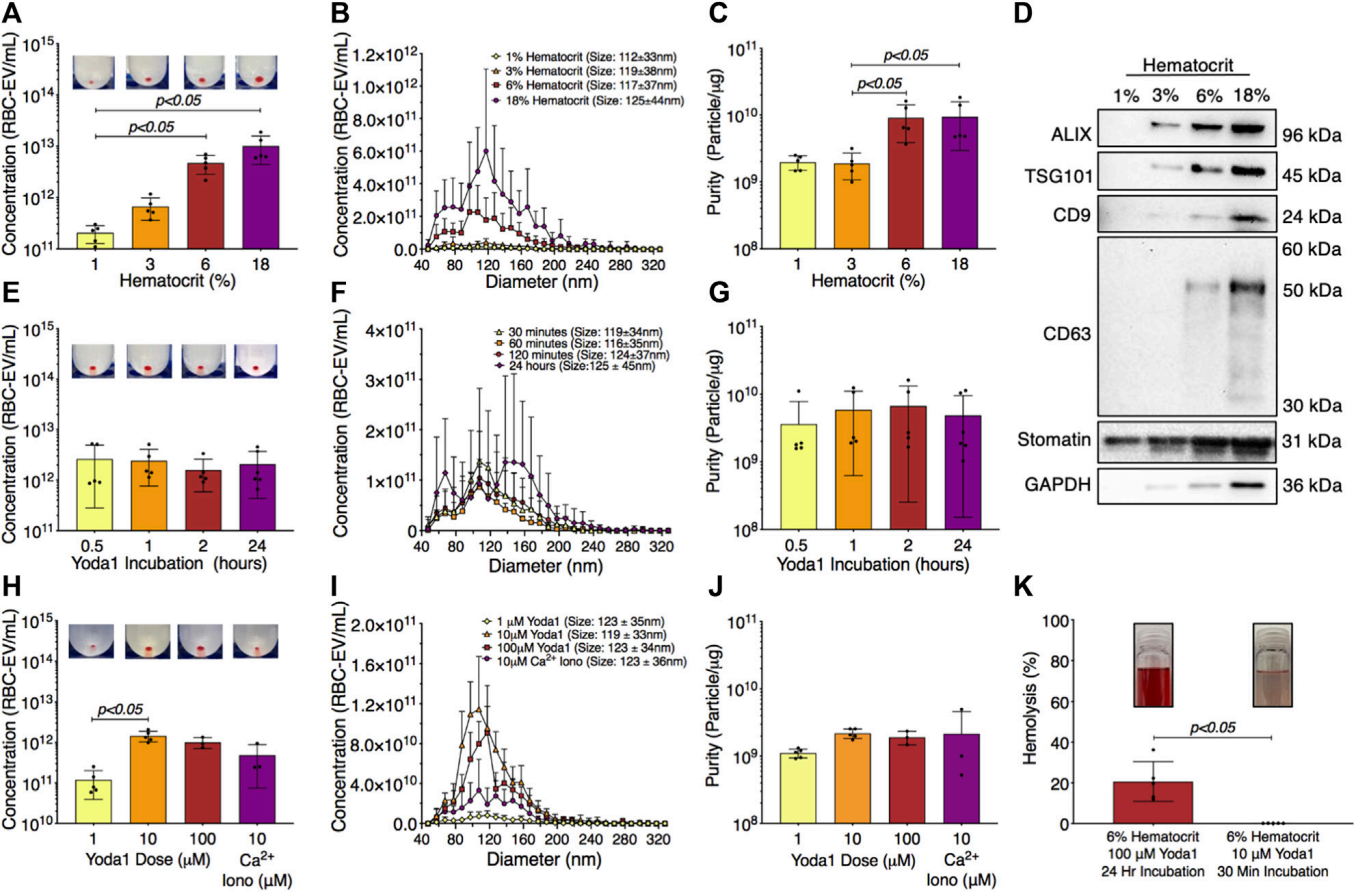
RBC-EVs generated using 6% hematocrit treated with 10 µM yoda1 for 30 min yielded the greatest RBC-EV concentration and purity while minimizing RBC hemolysis. **(A)** RBC-EV concentration (TRPS), **(B)** size histogram (TRPS), and **(C)** protein contamination (particle/µg protein) for 1, 3, 6, and 18% hematocrit treated with 100 µM yoda1 for 2 h **(D)** Representative Western blot of cytoplasmic EV markers ALIX and TSG101, surface EV markers CD9 and CD63, RBC-specific marker stomatin, and housekeeper GAPDH. **(E)** RBC-EV concentration (TRPS), **(F)** size histogram (TRPS), and **(G)** purity (particle/µg protein) for 6% hematocrit treated with 100 µM yoda1 for 0.5, 1, 2, and 24 h **(H)** RBC-EV concentration (TRPS) **(I)** size histogram (TRPS), and **(J)** purity (particle/µg protein) for 6% hematocrit treated with 1, 10, 100 µM yoda1 or 10 µM Ca^2+^ ionophore (iono) for 30 min. Inset in panels a, e, and h shows RBC-EV pellet after second ultracentrifugation at 100,000xg. **(K)** Hemolysis using original treatment (6% hematocrit, 100 µM yoda1, 24 h) and optimized treatment (6% hematocrit, 10 µM yoda1, 30 min) for RBC-EV production. Inset in panel k shows representative RBC supernatant to show hemolysis. Each data point represents a sample in which RBCs were pooled from four people. TRPS and purity studies were performed in singlicate and repeated five times (*n* = 5 per condition). Western blots were performed in triplicate and repeated two times (*n* = 6 per condition). Data are represented as mean ± standard deviation. Statistical significance was determined using Kruskal–Wallis with Dunn’s multiple comparison test for panels a, c, e, g, h, j and Mann-Whitney test for panel k.

RBC-EVs have been generated using a wide variety of protocols without a systemic study of how experimental parameters impact RBC-EV concentration and purity. Therefore, we determined the effect of hematocrit, yoda1 incubation time, and yoda1 concentration on piezo1 RBC-EV concentration, size, and purity. RBC-EV concentration increased with hematocrit. 1% hematocrit treated with yoda1 generated 2.1 × 10^11^ ± 7.7 × 10^10^ RBC-EV/mL, while 3% hematocrit produced 6.7 × 10^11^ ± 3.1 × 10^11^ RBC-EV/mL, 6% hematocrit produced 4.7 × 10^12^ ± 1.9 × 10^12^ RBC-EV/mL, and 18% hematocrit produced 1 × 10^13^ ± 5.7 × 10^12^ RBC-EV/mL (Figures 4A, B). While RBC-EV size was consistent across all hematocrits, RBC-EVs produced using 6% and 18% hematocrit had greater purity than RBC-EVs produced using 1% (*p* = 0.072 between 1% and 6% and *p* = 0.084 between 1% and 18%) and 3% hematocrit (*p* < 0.05; Figure 4C). We also detected cytoplasmic EV markers ALIX and TSG101, surface EV markers CD9 and CD63, and RBC-specific marker stomatin by Western blot in 6% and 18% hematocrit (Figure 4D).

We next tested different yoda1 treatment times to determine if RBC-EVs could be generated with shorter incubations. RBC-EVs ranging from 85 to 153 nm diameter were generated with only 30 min of yoda1 treatment (Figures 4E, F) and did not change in concentration, average size, or purity with up to 24 h of yoda1 treatment (Figures 4E, F). However, 24 h of yoda1 treatment generated three distinct RBC-EV size peaks at approximately 70, 110, and 150 nm diameter, while RBCs treated with yoda1 for 2 h or less generated one RBC-EV peak at 110 nm diameter (Figure 4F). Western blots also showed variability in molecular EV markers, particularly CD63, on piezo1 RBC-EVs generated using different hematocrit and yoda1 incubation times (Supplementary Figure S4). Since we did not observe increased EV generation or purity (Figure 4G) at longer incubation times, we used RBC treated with yoda1 for 30 min for subsequent experiments.

We then investigated how the yoda1 dose impacted RBC-EV generation. RBCs treated with 10 µM yoda1 produced significantly more RBC-EVs than RBCs treated with 1 µM yoda1, but 100 µM yoda1 did not produce more RBC-EVs than 10 µM yoda1 (Figures 4H, I). There was no difference in RBC-EV purity with 1, 10, 100 µM yoda1 or 10 µM Ca^2+^ ionophore treatment (Figure 4J). Based on these data, we determined that treatment of 6% hematocrit with 10 µM yoda1 for 30 min maximized RBC-EV generation and purity. The improved RBC-EV production method induced significantly less hemolysis than the original RBC-EV production method (6% hematocrit exposed to 24 h of 100 µM yoda1; Figure 4K).

### 3.5 RBC treatment conditions did not affect RBC-EV hemoglobin concentration

Hemoglobin composes 90% of the RBC proteome. Therefore, we studied how 6% hematocrit treated with 1) 10 µM yoda1 for 30 min, 2) 10 µM Ca^2+^ ionophore for 1 h, 3) 100 µM yoda1 for 24 h, and 4) 100 µM Ca^2+^ ionophore for 24 h affects RBC-EV hemoglobin concentration. We found that hemoglobin concentration increased linearly with RBC-EV particle concentration, regardless of the treatment condition (*R* ^2^ = 0.05; *p* < 0.05; Supplementary Figure S5).

### 3.6 Ultracentrifugation and ultrafiltration enabled reliable RBC-EVs with high purity

**FIGURE 5:**
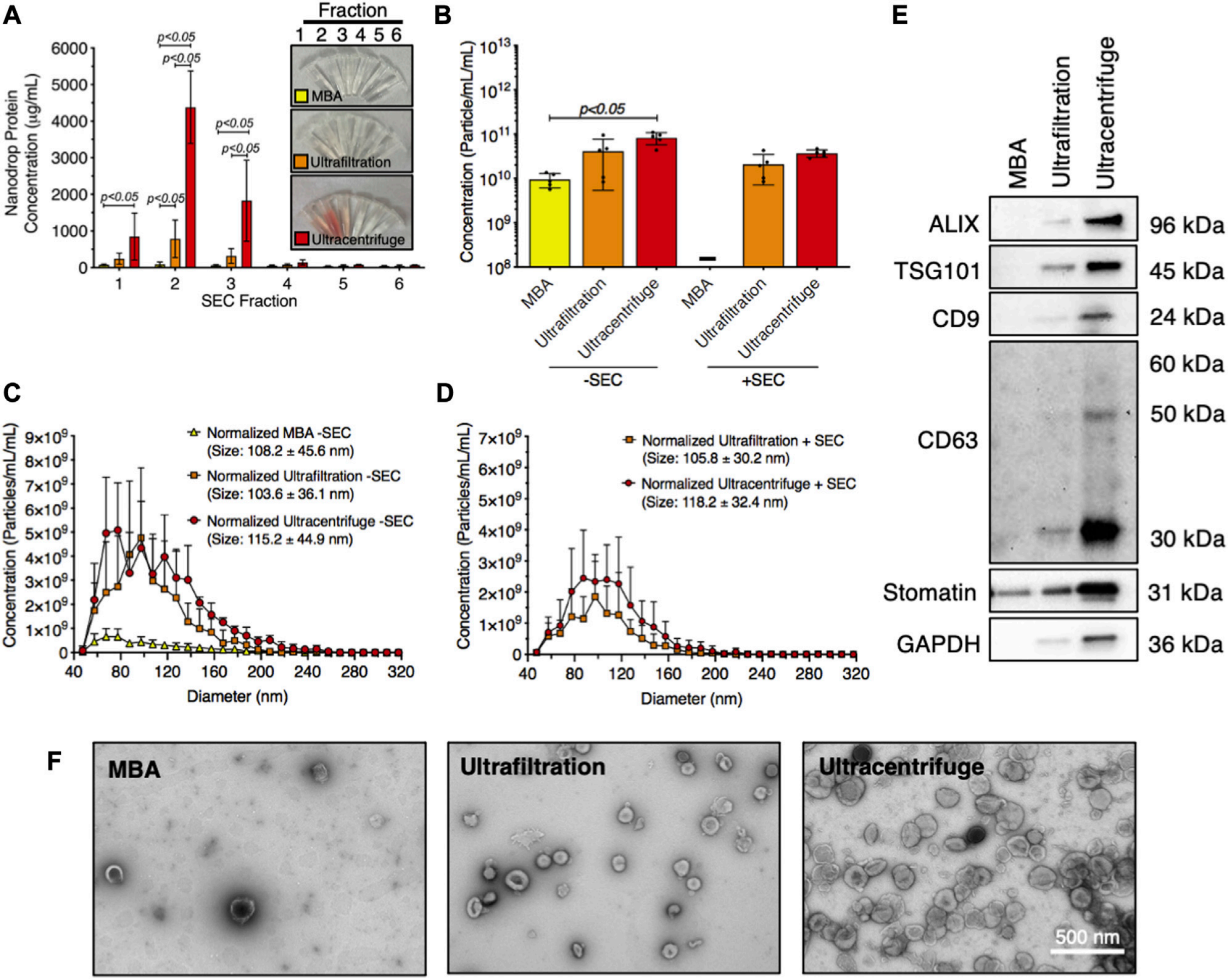
Piezo1 RBC-EVs were successfully isolated using ultracentrifugation and ultrafiltration. **(A)** Nanodrop protein measurements of SEC fractions from RBC-EVs isolated using membrane-based affinity (MBA), ultrafiltration, and ultracentrifugation. **(B)** Concentration (TRPS) of RBC-EVs isolated using membrane-based affinity, ultrafiltration, and ultracentrifugation with and without SEC purification. Concentration was normalized to the starting RBC supernatant volume. **(C)** RBC-EV size histogram (TRPS) isolated using membrane-based affinity, ultrafiltration, and ultracentrifugation *without* SEC purification. **(D)** RBC-EV size histogram (TRPS) isolated using ultrafiltration and ultracentrifugation *with* SEC purification. No RBC-EVs were detected in samples isolated using membrane-based affinity with SEC. **(E)** Representative Western blot of cytoplasmic EV markers ALIX and TSG101, surface EV markers CD9, and CD63, RBC-specific marker stomatin, apoptotic body marker calnexin, and housekeeper GAPDH. **(F)** TEM of RBC-EVs isolated using membrane-based affinity, ultrafiltration, and ultracentrifugation. For all experiments, RBCs (6% hematocrit) were treated with 10 µM yoda1 for 30 min “-” indicates that sufficient particles were not detected for TRPS concentration measurements. Each data point represents a sample in which RBCs were pooled from four people. Nanodrop and TRPS studies were performed in singlicate and repeated five times (*n* = 5 per condition). Western blots were performed in triplicate and repeated twice (*n* = 6 per condition). TEM images were acquired from two samples (*n* = 2 per condition). Data are represented as mean ± standard deviation. Statistical significance was determined using ordinary two-way ANOVA with Tukey’s multiple comparison test for panel a, and Kruskal–Wallis with Dunn’s multiple comparison test for panel b.

The technique used to isolate RBC-EVs can affect sample purity. Therefore, we compared the yield and purity of RBC-EVs isolated using membrane-based affinity binding (exoEasy), ultrafiltration (Amicon Ultra-15), and ultracentrifugation. We also assessed whether size exclusion chromatography (SEC) could further purify the RBC-EV samples. During SEC, samples were eluted into six 500 µL fractions. The highest protein concentrations were in the first three SEC fractions from ultrafiltration and ultracentrifugation samples, indicating that RBC-EVs were in these SEC fractions (Figure 5A). Protein levels were too low to identify RBC-EVs in any SEC fractions for samples isolated using membrane-based affinity, and no EVs were found by TRPS. Ultracentrifugation and ultrafiltration without SEC generated the most concentrated RBC-EVs (8.2 × 10^10^ ± 2.5 × 10^10^ and 4.1 × 10^11^ ± 3.5 × 10^10^ particle/mL per mL RBC-EV volume, respectively) compared to membrane-based affinity without SEC (*p* < 0.05; 9.4 × 10^9^ ± 3.3 × 10^9^ particle/mL per mL RBC-EV volume). SEC consistently decreased RBC-EV concentration (3.7 × 10^11^ ± 6.9 × 10^10^ particle/mL for ultracentrifugation with SEC, 2.3 × 10^11^ ± 1.2 × 10^10^ particle/mL for ultrafiltration with SEC; Figure 5B). RBC-EV isolation without SEC yielded particles 108.2 ± 45.6 nm in diameter using membrane-based affinity, 115.2 ± 44.9 nm in diameter using ultrafiltration, and 103.6 ± 36.1 nm in diameter using ultracentrifugation (Figure 5C). RBC-EV isolation with SEC did not significantly alter RBC-EV size (Figure 5D). All cytoplasmic and surface EV markers were detected by Western blot in RBC-EVs isolated using ultrafiltration and ultracentrifugation; however, EV markers were not detected from particles isolated using membrane-based affinity (Figure 5E). TEM verified our TRPS and Western blot results, revealing sparse EVs in samples isolated using membrane-based affinity, more EVs in samples isolated using ultrafiltration, and the most EVs in samples isolated using ultracentrifugation (Figure 5F).

**FIGURE 6:**
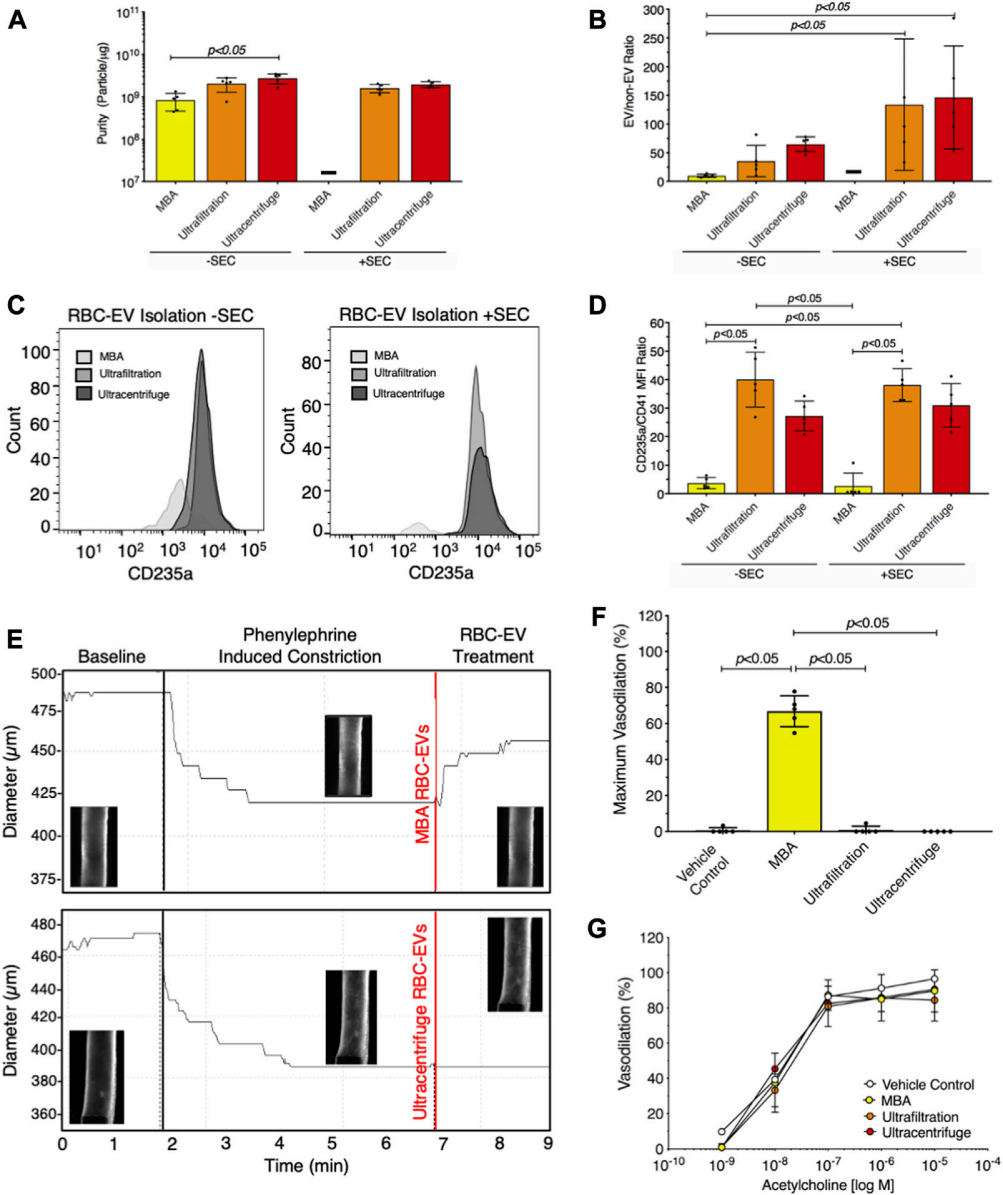
Ultracentrifugation produced the greatest RBC-EV yield and the lowest protein contamination. **(A)** Purity of RBC-EVs isolated using membrane-based affinity (MBA), ultrafiltration, and ultracentrifugation, measured by particle/µg protein. **(B)** Protein aggregates in RBC-EVs isolated using membrane-based affinity, ultrafiltration, and ultracentrifugation. EV/non-EV ratio was calculated by quantifying sample TRPS particle rate before and after lysing RBC-EVs using Triton-X 100. **(C)** Representative flow cytometry for RBC marker CD235a on RBC-EVs purified with and without SEC. **(D)** Platelet RBC-EV contamination for RBC-EVs isolated using membrane-based affinity, ultrafiltration, and ultracentrifugation with and without SEC, as quantified by the CD235a(RBC):CD41(platelet) ratio from flow cytometry. **(E)** Representative pressure myography data of phenylephrine constricted mouse carotid arteries treated with RBC-EVs. **(F)** Maximum mouse carotid artery vasodilation induced by RBC-EV treatment. **(G)** Endothelial-dependent mouse carotid artery vasodilation after 5 min RBC-EV treatment, measured using acetylcholine dose response. Controls included buffer XE (used to resuspend membrane-based affinity isolated RBC-EVs) and 2xPBS (used to resuspend ultrafiltration and ultracentrifuge isolated RBC-EVs). “-” indicates that sufficient particles were not detected for TRPS concentration measurements. Each data point represents a sample in which RBCs were pooled from four people. All studies were performed in singlicate and repeated five times (*n* = 5 per condition). Data are represented as mean ± standard deviation. Statistical significance for panels a, b, d, and f was determined using Kruskal–Wallis with Dunn’s multiple comparison test.

We then compared RBC-EV isolation methods for yield and purity by measuring RBC-EV protein contamination. Particles isolated using membrane-based affinity were less pure (8.2 × 10^8^ ± 3.8 × 10^8^ particles/µg protein) than RBC-EVs isolated by ultracentrifugation 2.8 × 10^9^ ± 7.3 × 10^8^ particles/µg; *p* < 0.05; Figure 6A). SEC did not improve purity in RBC-EVs isolated using ultrafiltration or ultracentrifugation (Figure 6A). Protein aggregates were measured using EV/non-EV particle rate ratio, calculated by quantifying the sample particle rate before and after lysing RBC-EVs with Triton-X 100. EV/non-EV particle rate ratio suggested no statistically significant difference in protein aggregate contamination amongst membrane-based affinity, ultrafiltration, and ultracentrifugation RBC-EV isolation methods (Figure 6B). SEC decreased RBC-EV protein aggregates 2.3-fold after ultracentrifugation and 3.8-fold after ultrafiltration isolation, but the results were variable amongst the repeated expriments (Figure 6B). Flow cytometry did not show a visible rightward shift in CD235a positive peak after RBC-EVs were passed through SEC (Figure 6C; Supplementary Figure S6), suggesting that SEC did not significantly enhance RBC-EV purity. SEC also did not significantly decrease platelet contamination in EV samples isolated using ultrafiltration or ultracentrifugation (Figure 6D; Supplementary Figure S7). However, RBC-EVs isolated by ultracentrifugation contained significantly less platelet EV contamination (CD41^+^) than particles isolated using membrane-based affinity, as measured by flow cytometry (Figure 6D).

To determine if RBC-EV purity impacted functional assays, we studied how RBC-EVs isolated using membrane-based affinity, ultrafiltration, and ultracentrifugation affected mouse carotid artery vasodilation. Pre-constricted mouse carotid arteries treated with membrane-based affinity-isolated RBC-EV rapidly vasodilated 66% ± 8.6%, while ultrafiltration- and ultracentrifuge-isolated RBC-EVs did not cause vasodilation (Figures 6E, F). Carotid arteries treated with RBC-EV solvents 2xPBS (used to resuspend RBC-EVs isolated using ultrafiltration and ultracentrifugation) and buffer XE (used to elute RBC-EVs isolated via membrane-based affinity) did not cause vasodilation. Endothelial-dependent vasodilation, measured by acetylcholine dose response in mouse carotid arteries, did not significantly change with RBC-EV or vehicle control treatment (Figure 6G). Untreated carotid arteries maximally vasodilated 96.5% ± 3.2%, while membrane-based affinity, ultrafiltration, and ultracentrifugation piezo1 RBC-EV treated carotid arteries maximally vasodilated 89.7% ± 12.0%, 84.4% ± 12.0%, and 90.6% ± 11.7% respectively (Figure 6G).

### 3.7 RBC-EVs isolated from fresh and frozen RBC supernatant had similar yield, size, and purity

Finally, we investigated how storage affects RBC-EV concentration, size, and purity. First, we isolated RBC-EVs from yoda1-treated RBC supernatant that was either fresh or had undergone one freeze-thaw cycle (−80°C to 25°C). We found no significant differences in EV concentration, purity, or size (Supplementary Figures S8A–C) between RBC-EVs isolated using fresh or frozen RBC supernatant. We then subjected RBC-EVs isolated from fresh RBC supernatant to one, two, or three freeze-thaw cycles. RBC-EVs that underwent 1 freeze-thaw cycle contained 2.4 × 10^12^ ± 2.3 × 10^11^ RBC-EV/mL at 109 ± 31 nm diameter, similar to the concentration and size prior to the freeze-thaw cycle. However, RBC-EV concentration decreased by 79.2% and size decreased by 32.3% after the second freeze-thaw cycle (*p* < 0.05; Supplementary Figures S8D–F). Moreover, RBC-EVs stored at 4°C decreased concentration by 58.3% and size by 30.8% over 7 days (*p* < 0.05; Supplementary Figures S8G–I). These data suggest smaller RBC-EVs are more stable during multiple freeze-thaw cycles and 4°C storage.

## 4 Discussion

RBC-EVs have primarily been studied in pathological conditions, and a wide variety of methods have been used to generate, isolate, and characterize EVs. RBC-EVs generated under physiological conditions, for example, by shear stress, are also of interest because they may contribute to the circulating EV pool in healthy individuals; however, the lack of standard ways to generate, isolate, and store shear stress-generated RBC-EVs hinders mechanistic studies. We now show that both shear stress and piezo1 stimulation trigger RBC-EV release. Treatment of 6% hematocrit with 10 µM yoda1 in the presence of Ca^2+^ generated high piezo1 RBC-EV concentrations within 30 min. RBC-EVs were effectively isolated using either ultrafiltration or ultracentrifugation without the need for further purification by SEC. RBC-EVs were isolated from fresh or frozen RBC supernatant after yoda1 treatment without significantly altering RBC-EV size or concentration. Isolated piezo1 RBC-EVs samples should be stored at −80°C and used immediately after thawing, as multiple freeze-thaw cycles and storage at 4°C decreased RBC-EV size and concentration. These methods can be implemented by researchers studying RBC-EVs to further our understanding of physiological and pathological RBC-EVs.

Our data show that RBC-EVs circulate in healthy humans, suggesting a physiological function for RBC-EVs. Nemkov *et al.* also showed that healthy individuals have circulating RBC-EVs when they detected <1 µm diameter particles with both RBC marker CD235a and EV marker phosphatidylserine by flow cytometry (Nemkov et al., 2021). Moreover, they found that 30 min of high-intensity cycling may increase RBC-EV shedding (Nemkov et al., 2021), potentially due to augmented capillary perfusion that increases RBC shear stress exposure. These data suggest that healthy RBCs may release EVs in response to altered vascular hemodynamics, as seen during exercise, to support vascular function.

We further show that exposing RBCs to shear stress via a rheometer or mechanically stimulating RBCs via piezo1 generates RBC-EVs. Our methods are simpler, faster, and easier to control than prior efforts that generated EVs by shearing RBCs through a syringe (Jana et al., 2018). Since RBCs begin to deform around 5–20 dynes/cm^2^ and rupture around 1,500 dynes/cm^2^ (Leverett et al., 1972; Dupire et al., 2012), our method of applying 250 dynes/cm^2^ (25 Pa) generates EVs via RBC deformation and not rupture. We also observed <1% hemolysis with RBC shear stress exposure, further confirming that shear-stress-generated RBC-EVs were not formed by rupture. The presence of ALIX, TSG101, CD9, and CD63 suggests that RBCs contain complex mechanisms that sort and package proteins into piezo1 RBC-EVs (Thangaraju et al., 2020; Gurung et al., 2021). Therefore, mechanical and piezo1 stimulation may package beneficial biomolecules into RBC-EVs that are then systemically transported.

Shear stress may trigger RBCs to release EVs via piezo1 stimulation. RBC piezo1 opens in response to mechanosensitive stimuli, such as shear stress, which then increases Ca^2+^ entry into the cell (Cahalan et al., 2015). Increasing intracellular RBC Ca^2+^ using Ca^2+^ ionophore is the standard method to generate RBC-EVs (Allan et al., 1980; Salzer et al., 2002; Nguyen et al., 2016; Usman et al., 2018; Bigdelou and Farnoud, 2020). Interestingly, RBC Ca^2+^ uptake via piezo1 stimulation led to similar RBC-EV generation with less RBC death, hemolysis, and cell debris than Ca^2+^ ionophore treatment. The presence of EV markers ALIX, TSG101, CD9, and CD63 in RBCs and RBC-EVs, and RBC-specific markers stomatin and CD235a in RBC-EVs suggests that the piezo1 RBC-EVs were released by RBCs rather than other circulating cells such as leukocytes, platelets, and reticulocytes. Together, these data suggest that piezo1 stimulation via shear stress or yoda1 may induce a more controlled Ca^2+^ influx or activate alternative RBC mechanotransduction pathways than Ca^2+^ ionophore treatment.

*In vitro* RBC-EV production methods should recapitulate the way in which the EVs are produced *in vivo,* as production conditions can impact EV yield and contents (Coumans et al., 2017). RBC treatment time is particularly important since RBCs will eventually consume available glucose, leading to non-physiological RBC-EV release due to membrane disruption from adenosine triphosphate (ATP) depletion (Almizraq et al., 2018; Cloos et al., 2020). Currently, RBC-EVs meant to simulate *in vivo* EVs are generated *in vitro* using treatment times ranging from 2 to 48 h (Almizraq et al., 2018; Noulsri and Palasuwan, 2018; Peng et al., 2020), whereas RBC-EVs meant to simulate storage RBC-EVs are generated over several weeks (Almizraq et al., 2018; Noulsri and Palasuwan, 2018). In this study, we found that treating as little as 1 mL RBCs for as low as 30 min generated ∼10^11^ RBC-EVs with low hemolysis and high EV purity. Given that the total circulating EV concentration is ∼10^10^ particles/mL, this method generates enough EVs for biological studies (Johnsen et al., 2019). Interestingly, we found that 24-h yoda1 treatment generated RBC-EVs with three distinct size peaks, compared to only one peak for RBC-EVs generated in less than 2 h. Piezo1 stimulation increases ATP release from human RBCs (Cinar et al., 2015), thus long-term piezo1 stimulation may accelerate ATP depletion. Therefore, long-term piezo1 stimulation may generate EV subpopulations similar to those generated by RBC storage.

EV isolation methods can similarly impact EV yield and purity. We found that ultracentrifugation provided the greatest and most consistent piezo1 RBC-EV yield but was the most time-consuming method (∼3 h). Ultrafiltration was faster and more accessible, requiring only 1 h in a benchtop centrifuge. Ultrafiltration was successfully used to isolate both piezo1 and shear stress-generated RBC-EVs. However, ultrafiltration caused greater variability in piezo1 RBC-EV yield than ultracentrifugation (Figure 5B). Membrane-based affinity was also faster and more accessible than ultracentrifugation but yielded lower particle concentrations than both ultrafiltration and ultracentrifugation. This low yield made characterizing any RBC-EVs present in these samples challenging. We also found that particles isolated using membrane-based affinity contained more protein, protein aggregates, and platelet EVs than particles isolated using ultracentrifugation or ultrafiltration. For example, particles isolated using membrane-based affinity after RBC piezo1 stimulation (Figure 5E) contained stomatin, which is an RBC membrane protein, but did not contain EV markers ALIX, TSG101, or CD9. We therefore postulate that the stomatin was in non-EV particles released by RBCs such as protein aggregates. Our data finally show that ultracentrifugation and ultrafiltration are sufficient to separate RBC-EVs from protein contamination, as 1) SEC did not significantly diminish protein and platelet EV contamination and 2) piezo1 and Ca^2+^ ionophore RBC-EVs had similar purities, even though Ca^2+^ ionophore treatment produced more hemolysis and subsequent hemoglobin contamination than yoda1 treatment.

In this study, RBC-EVs isolated using ultracentrifugation contained particle/total protein ratios of ∼10^9^ particles/µg, 10x higher than EVs isolated from plasma or serum EVs (Serrano-Pertierra et al., 2019; Brennan et al., 2020). Webber *et al.* proposed particle/total protein criteria to define EV purity, suggesting that ratios > 3 × 10^10^ particles/µg equate to high EV purity, ratios from 2 × 10^9^ to 2 × 10^10^ particles/µg equate to low purity, and any ratio below 1.5 × 10^9^ particles/µg to be considered impure. However, as noted by the authors, their particles/µg calculations may overestimate the true number of vesicles isolated, since they assumed that all detected particles were vesicles and thus did not account for protein aggregates and other large contaminates. Additionally, particles/µg purity measurements assume that all EVs contain similar protein concentrations. In reality, EV protein concentration likely depends on the originating cell type and EV generation conditions.

The importance of RBC-EV purity for biological studies is highlighted by our pressure myography studies. Particles isolated from piezo1 RBCs using membrane-based affinity caused rapid mouse carotid artery vasodilation; however, particles isolated by ultrafiltration and ultracentrifugation, which were purer, did not cause vasodilation. Thus, it is likely that protein contaminants in the particles isolated by membrane-based affinity triggered vasodilation. Piezo1 stimulation has been shown to trigger RBCs to produce and release vasodilatory biomolecules, such as ATP and nitric oxide (Cinar et al., 2015; Kuck et al., 2022). Forteza-Genestrla *et al.* similarly highlighted the importance of EV purity. They found that mesenchymal stromal cell EVs isolated using ultracentrifugation (120,000xg for 18 h) increased metalloproteinases and decorin and decreased collagen and aggrecan mRNA levels in mouse chondrogenic cells (Forteza-Genestra et al., 2020). These pathological effects, however, were mitigated when the EVs were further purified using SEC (Forteza-Genestra et al., 2020). These data highlight the need to check EV purity and characterize the impact of non-EV contamination in biological studies.

Our data on piezo1 RBC-EV stability in storage may have implications for storage RBC-EVs. Other studies reported high RBC-EV concentrations following RBC storage at 4°C for several weeks (Rubin et al., 2008; Tzounakas et al., 2016; Hashemi Tayer et al., 2019). RBC-EVs secreted from stored RBCs increased procoagulant activity, clotting time, and vascular dysfunction through heme-mediated endothelial dysfunction and reduced nitric oxide bioavailability (Donadee et al., 2011; Liu et al., 2013). Additional research suggested that EVs in stored blood contribute to adverse transfusion reactions, including transfusion-related acute lung injury and transfusion-associated circulatory overload (Wannez et al., 2019; Yoshida et al., 2019). Our data suggesting that smaller EVs are more stable in storage could indicate that these smaller EVs have different contents that are more damaging to the vasculature than larger EVs. RBC storage for transfusions may also impair mechanosensitive RBC mechanisms by depleting ATP or through the stress caused by freeze-thaw cycles, thus reducing beneficial RBC-EVs produced in physiological flow after transfusion.

Our studies focused on the RBC-EVs produced during physiological mechanical stresses; however, RBC-EV production may also increase in pathological conditions in which RBC shear stress increases. For example, RBCs experience higher than normal shear stress when they squeeze through a constriction in a patient with coarctation of the aorta, a stenosed mitral valve, or an obstructive atherosclerotic plaque. Additionally, implanted artificial heart valves and circulatory assist devices (e.g., left ventricular assist devices), as well as perfusion devices used during dialysis or extracorporeal membrane oxygenation also apply stresses to RBCs that could induce mechanical EV production. Moreover, physiological flow-induced mechanosensitive EV generation is likely altered by diseases that affect RBC (e.g., sickle cell disease), cardiovascular disease risk factors (e.g., obstructive sleep apnea, diabetes), and RBC storage. For example, circulating RBC-EVs increase in patients with diabetes (Freeman et al., 2018). Future work examining the production and contents of RBC-EVs produced in various mechanostimulated environments will provide novel insights into how RBC-EVs affect human health and disease.

While our study describes methods to produce, isolate, and store mechanically-generated RBC-EVs, it is not without limitations. First, TRPS measurements require particles to pass through a physical nanopore, which limits particle characterization to a narrow size range. As a result, our study quantified EVs ranging in size from 50-220 nm, as we used a 0.22 µM syringe filter to prevent large particles from blocking the nanopore. It is likely that RBC piezo1 stimulation and shear stress trigger the release of EVs up to 1,000 nm in diameter, which we did not characterize in the present study. We also did not compare circulating, shear stress-induced, piezo1, and Ca^2+^ ionophore RBC-EV contents, as this work aims to establish methods to generate physiologically-relevant piezo1 RBC-EVs. Moreover, RBC-EVs contain diverse biochemical contents, which likely vary from one person to another. This is supported by our Western blot data that show variability in molecular RBC-EV markers generated from the same donors on different days (Supplementary Figure S4). Additional studies using our methods are needed to understand how piezo1 RBC-EV contents change when they are generated by different stimuli.

## 5 Conclusion

We now provide methods to produce RBC-EV via shear stress and piezo1 stimulation, as well as isolate, characterize, and store these RBC-EV. Piezo1 stimulation of 6% hematocrit with 10 µM yoda1 for 30 min preserved RBC viability, induced less hemolysis, and produced enough RBC-EVs for mechanistic studies from just 1 mL of blood. Ultracentrifugation resulted in the greatest RBC-EV yield and purity, but ultrafiltration required the least time and still provided sufficient RBC-EV yield and purity. This piezo1 RBC-EV production and isolation protocol will enable biological studies to show how RBC-EVs generated via physiological shear stress affect vascular health.

## Data Availability

The raw data supporting the conclusion of this article will be made available by the authors, without undue reservation.
