# Supplementary material for: Mechanical stimuli such as shear stress and piezo1 stimulation generate red blood cell extracellular vesicles
Source: Front Physiol. 2023 Aug 30;14:1246910. doi: 10.3389/fphys.2023.1246910 (PMC10502313; doi:10.3389/fphys.2023.1246910)
Supplement: Supplementary file 1 [file DataSheet1.pdf]

Supplementary Material

Mechanical Stimuli such as Shear Stress and Piezo1 Stimulation Generate Red Blood Cell Extracellular Vesicles

Gurneet S. Sangha[1], Callie M. Weber[1], Ryan M. Sapp[1], Saini Setua[2], Kiruphagaran Thangaraju[2], Morgan Pettebone[1], Stephen C. Rogers[2], Allan Doctor[2], Paul W. Buehler[2,3], Alisa M. Clyne[1*]

[1] Fischell Department of Bioengineering, University of Maryland, College Park, MD, USA

[2] Center for Blood Oxygen Transport and Hemostasis, Department of Pediatrics, University of Maryland School of Medicine, Baltimore, MD, USA

[3] Department of Pathology, University of Maryland School of Medicine, Baltimore, MD, USA

*** Correspondence:**
Alisa. M. Clyne
aclyne@umd.edu

1 Supplementary Figures

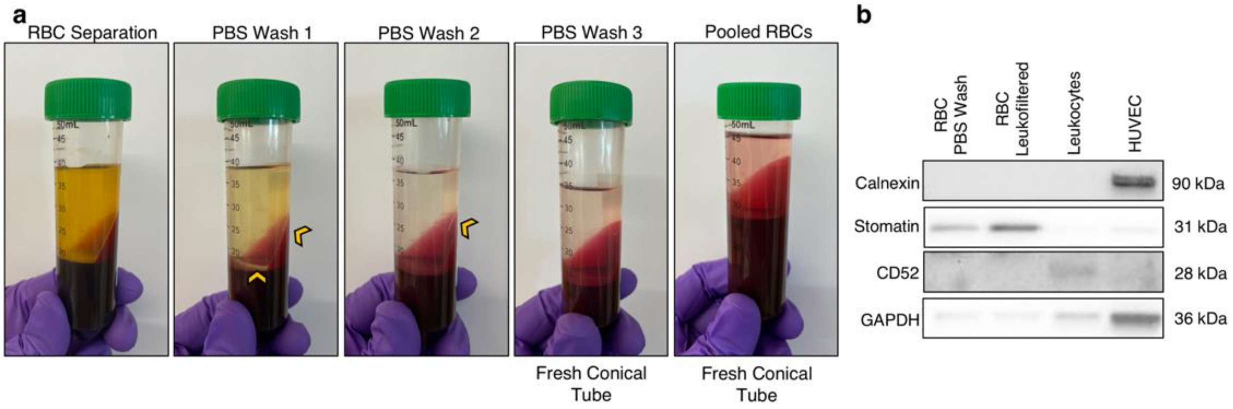

Supplementary Figure 1 | Triple washing RBCs in PBS sufficiently removed leukocytes and other large cells. a. Images illustrating how RBCs were washed and isolated from plasma and buffy coat. RBCs were washed until contamination (yellow arrow) was removed. **b.** Western blot showing that PBS washes and leukofilters removed leukocytes (CD52) and other cell types containing endoplasmic reticulum (calnexin).

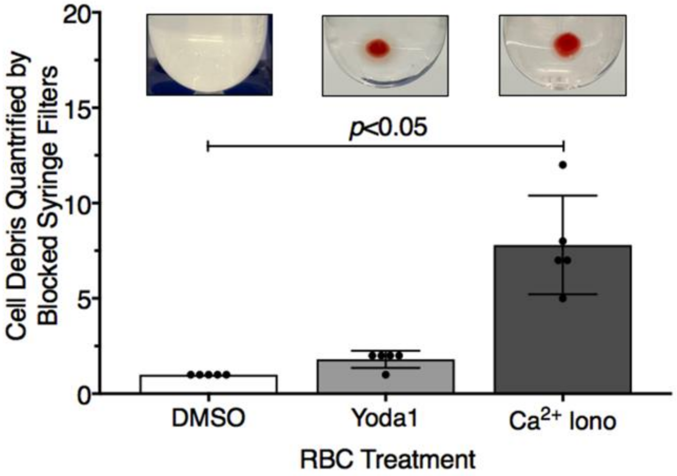

Supplementary Figure 2 | Ca^{2+} ionophore treatment generated more cell debris than yoda1 treatment. RBC debris after 0.28% DMSO (vehicle control), 10 µM yoda1, and 10 µM Ca^{2+} ionophore treatment for 30 minutes, as quantified by the number of syringe filters that were 100% blocked when filtering RBC supernatant. RBC supernatant was centrifuged at 600, 1600, 3260, and 10,000xg to decrease cell debris prior to syringe filtering. Inset shows representative image of RBC debris pellet after 10,000xg centrifugation. Each data point represents a sample in which RBCs were pooled from four different people. This experiment was performed in singlicate and repeated five times (n=5 per condition). Data represented as mean ± standard deviation. Statistical significance was determined using Kruskal-Wallis with Dunn's multiple comparison test.

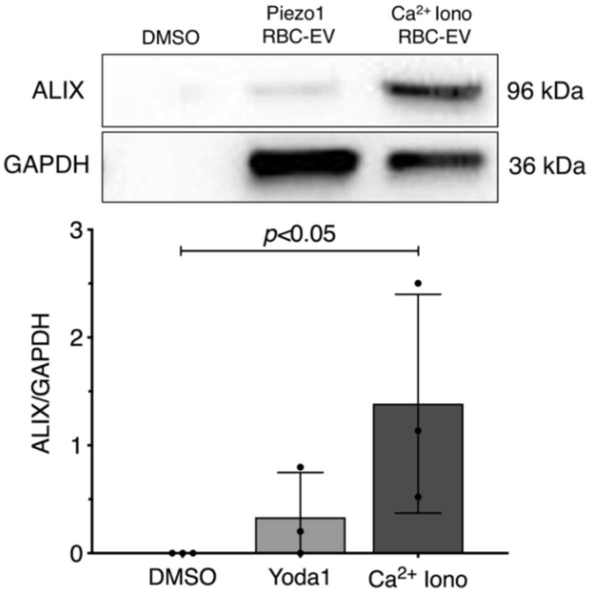

Supplementary Figure 3 | Ca^{2+} ionophore RBC-EVs contained more ALIX than piezo1 RBC-EVs. Western blots (9.75 µL sample/well) of cytoplasmic EV markers ALIX and housekeeper GAPDH in particles released after DMSO treatment (vehicle control) and in Piezo1 and Ca^{2+} ionophore RBC-EVs. Western blot shows one representative comparison out of three total comparisons. Data represented as mean ± standard deviation. Statistical significance was determined using Kruskal-Wallis with Dunn's multiple comparison test.

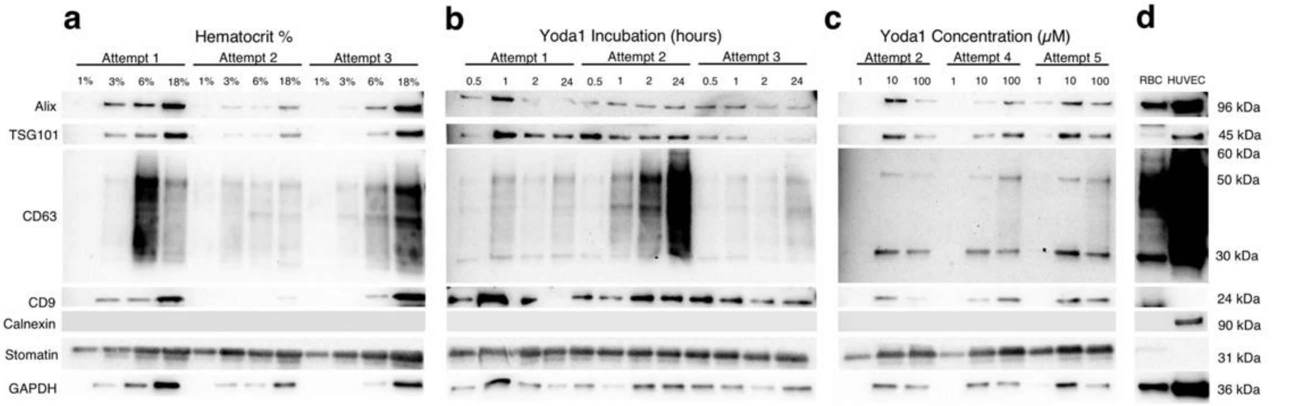

Supplementary Figure 4 | RBC-EV molecular markers varied depending on treatment conditions, blood donors, and biological replicate. Western blots (9.75 µL sample/well) for RBC-EV marker (Alix, TSG101, CD63, and CD9), RBC-specific (Stomatin), and apoptotic body marker (Calnexin) in **a.** hematocrit dose response experiments, **b.** yoda1 timecourse experiments, **c.** yoda1 dose response experiments, and **d.** RBCs and human umbilical vein endothelial cells (HUVEC) lysates. Western blots show markers on RBC-EVs isolated on three different days. For each experiment, RBC-EVs were isolated from RBCs pooled from three different donors.

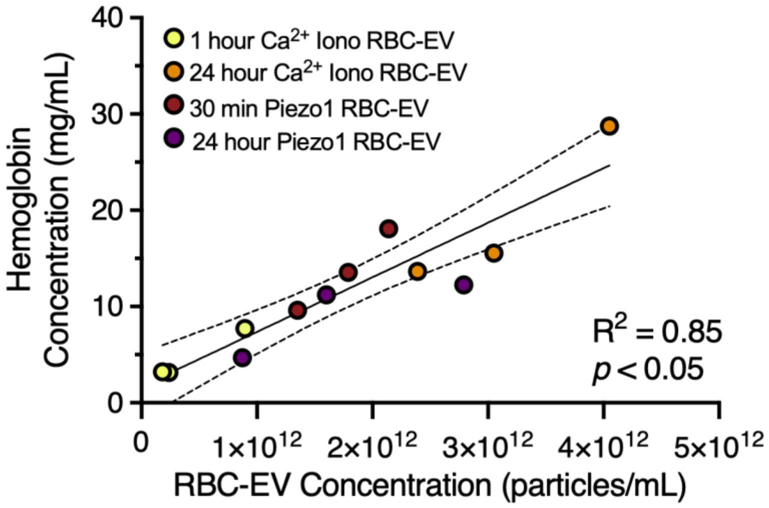

Supplementary Figure 5 | Hemoglobin concentration increases linearly with increasing RBC-EV concentration. Hemoglobin ELISA showed that samples with greater RBC-EV concentration contained greater hemoglobin concentration. RBC-EVs were generated by treating 6% hematocrit with 1) 10 µM yoda1 for 30 minutes, 2) 10 µM Ca^{2+} ionophore for 1 hour, 3) 100 µM yoda1 for 24 hours, and 4) 100 µM Ca^{2+} ionophore for 24 hours.

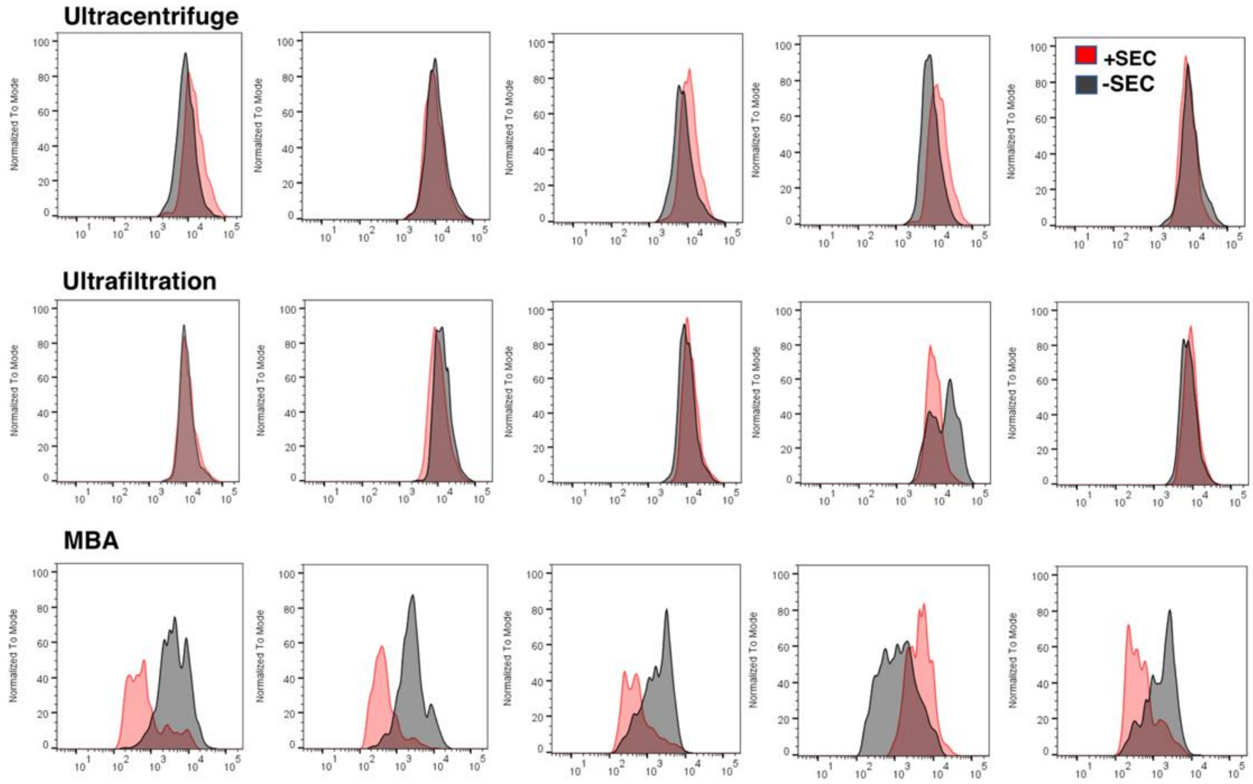

Supplementary Figure 6 | SEC did not significantly improve RBC-EV purity. Flow cytometric analysis of RBC-EVs (identified by RBC marker CD235a), isolated using ultracentrifugation (n=5), ultrafiltration (n=5) and membrane-based affinity (n=5) with and without SEC. We did not observe a rightward shift of the RBC-EV peaks, suggesting that SEC did not purity sample.

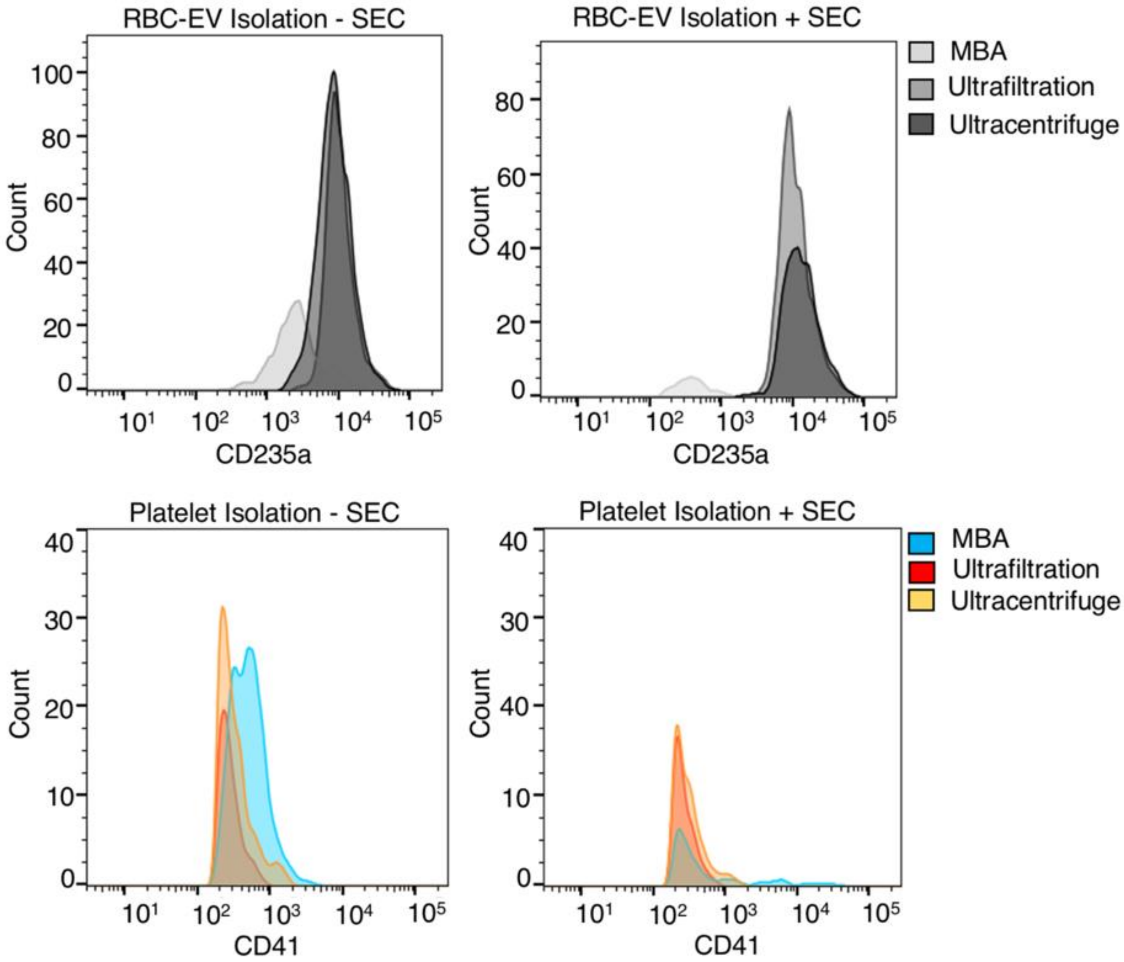

Supplementary Figure 7 | SEC did not significantly decrease platelet-EV contamination. Representative flow cytometric analysis of RBC-EVs (identified by RBC marker CD235a) and platelet-EVs (identified by platelet marker CD41), isolated using MBA, ultrafiltration, and ultracentrifugation with and without SEC. We did not observe a significant rightward shift of the RBC-EVs, suggesting that SEC did not purify samples. The relevant count of platelet EVs decreases for all isolation methods, although not significantly.

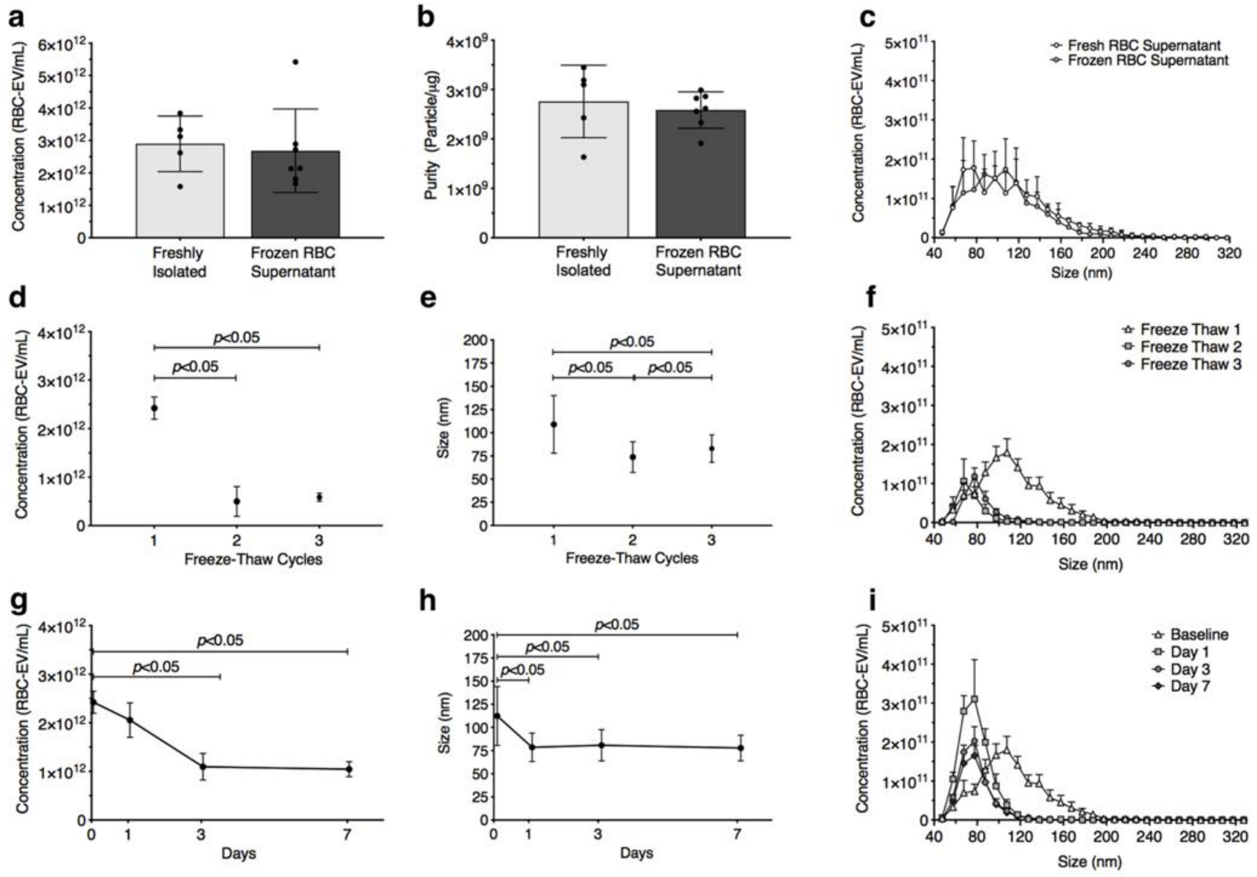

Supplementary Figure 8 | RBC-EV concentration and size changed with storage conditions. a. Concentration, **b.** purity, and **c.** size histogram of RBC-EVs isolated using fresh RBC supernatant and frozen RBC supernatant. **d.** Concentration, **e.** average size, and **f.** size histogram of RBC-EVs after up to 3 freeze thaw cycles. **g.** Concentration, **h.** average size, and **i.** size histogram of RBC-EVs stored in 4ºC for up to 7 days. Size and concentration were measured using TRPS and purity was measured by calculating particle/total protein ratio. All samples were generated by treating 6% hematocrit with 10 µM yoda1 for 30 minutes. Size, concentration, and purity studies for RBC-EVs isolated from fresh and frozen RBC supernatant were performed in singlicate and repeated five to seven times (n=5-7 per condition). Freeze-thaw cycle and time course studies were performed in singlicate and repeated five times (n=5 per condition). Data represented as mean ± standard deviation. Statistical significance for panels d, e, g, and h was determined using Kruskal-Wallis with Dunn's multiple comparison test.
